# How the Addition of Chitosan Affects the Transport and Rheological Properties of Agarose Hydrogels

**DOI:** 10.3390/gels9020099

**Published:** 2023-01-23

**Authors:** Martina Klučáková

**Affiliations:** Faculty of Chemistry, Brno University of Technology, Purkyňova 118, 612 00 Brno, Czech Republic; klucakova@fch.vutbr.cz

**Keywords:** chitosan, copper, diffusion, Liesegang pattern, solubility product, immobilization

## Abstract

Agarose hydrogels enriched by chitosan were studied from a point of view diffusion and the immobilization of metal ions. Copper was used as a model metal with a high affinity to chitosan. The influence of interactions between copper and chitosan on transport properties was investigated. Effective diffusion coefficients were determined and compared with values obtained from pure agarose hydrogel. Their values increased with the amount of chitosan added to agarose hydrogel and the lowest addition caused the decrease in diffusivity in comparison with hydrogel without chitosan. Liesegang patterns were observed in the hydrogels with higher contents of chitosan. The patterns were more distinct if the chitosan content increased. The formation of Liesegang patterns caused a local decrease in the concentration of copper ions and concentration profiles were affected by this phenomenon. Thus, the values of effective diffusion coefficient covered the influences of pore structure of hydrogels and the interactions between chitosan and metal ions, including precipitation on observed Liesegang rings. From the point of view of rheology, the addition of chitosan resulted in changes in storage and loss moduli, which can show on a “more liquid” character of enriched hydrogels. It can contribute to the increase in the effective diffusion coefficients for hydrogels with higher content of chitosan.

## 1. Introduction

Chitosan is a hydrophilic polyelectrolyte obtained by the deacetylation of chitin, which is widely spread among marine and terrestrial invertebrates and in lower forms of the plant kingdom. It has unique properties as biocompatibility, bioactivity, and biodegradability [1,2,3,4]. It has low toxicity, hemostatic potential, and a good ability to form films [5,6,7,8]. It can be used in many industrial and biomedical applications, e.g., [9,10,11,12,13,14,15]. Chitosan is one of the most efficient adsorbents, receiving considerable interest for heavy metals removal due to its very good adsorption capacities and relatively low cost [4,7,8,16]. Its adsorption properties are attributed to high hydrophilicity (due to OH groups), primary amino groups with high activity, and flexible structure of polymer chains [17]. Major sources of heavy metals in nature are mainly from industry, mining, metal plating, corrosion, and electronic device manufactures. Waste streams from these workings may contain considerable amount of toxic and polluting metal ions. Copper is one of the most widespread metal contaminants in nature [8]. Copper is extensively used in the electrical industry, production of fungicides, and anti-fouling paints. Additionally, it is an essential trace element for people, although it can be harmful when a large dosage is ingested [4,18,19]. It is not biodegradable and can accumulate in living organisms. It can cause various toxicological effects, e.g., gastrointestinal irritation, hypertension, sterility, and intellectual disability [19,20,21]. The adsorption of metal ions is a widely used method for removing metal ions from nature. The use of chitosan is also widely used for these purposes [16,19,21,22,23,24,25], although its adsorption capacity for copper shows significant differences among published studies [16]. Chitosan can be physically and chemically modified to improve their mechanical properties [4]. They can be used in the forms of hydrogel beads [4,16,18], resin [26], nanoparticles [12], and membranes [8]. Detail analysis of adsorption of copper (and other metal ions) on chitosan and chitosan-based materials can be found in [22,23,24,25,27].

As mentioned above, many authors deal with chitosan as the material has a good affinity with different substances, including metal ions. However, published papers are mainly focused on the adsorption and the use of chitosan as an important constituent of adsorbents. The studies on the mobility of metal ions and diffusion processes in chitosan materials are scarce. Some authors observed an influence of intraparticle diffusion on the adsorption kinetics of metal ions on chitosan [4,28,29,30,31]. In general, the adsorption can include additional steps of external and intraparticle diffusion: film or external diffusion, pore diffusion, and surface diffusion. The external diffusion is usually not the major limiting step in the adsorption, so the attention is mainly focused on simple intra-particular diffusion models [4,27,28,29]. All of the above-mentioned approaches relate to the adsorption processes. The diffusion in chitosan materials as the main topic is studied only in several works [32,33,34]. In ref. [32], chitosan membranes were prepared, then characterized from point of view of the permeation of KCl. Concentration changes in the donor and acceptor compartments were determined on the basis of conductivity measurements. It means that measured values of conductivity included influence of all present ions. No diffusion coefficient was calculated. It was stated that results can be influenced by changes in the membrane structure during experiments and the occurrence of osmosis. Krajewska et al. [33] studied diffusion of 15 metal ions through several types of hydrogel chitosan membranes. The diffusive permeability coefficients of metal ions were strongly influenced by the treatment of membrane. This value was determined as 1.8 × 10^−11^ m^2^ s^−1^ for the diffusion through pure chitosan membrane, and decreased for the membrane treated by glutaraldehyde to 1.6 × 10^−12^ m^2^ s^−1^. The permeability coefficient is a product of the solute solubility coefficient in the membrane (a dimensionless number expressed by ratio of solute content in membrane and in solution), and the solute diffusion coefficient. The salt diffusive permeability coefficients were obtained from the recorded steady states of salt diffusion through the membranes. It was found that, except for the diffusion of Cu(II) and Ni(II) ions through chitosan membranes, the steady states were established within a short time. For the diffusion of Cu(II) and Ni(II) through chitosan membranes, time lags between 10 and 20 min were observed. These results were attributed to high affinity of chitosan to these metal ions.

Yoshida et al. [34] dealt with the diffusion of potassium sorbate through chitosan and palmitic acid chitosan films. They obtained diffusion coefficient in the range from 1.22 × 10^−13^ to 1.16 × 10^−12^ m^2^ s^−1^ in the dependence of membrane type, and used a mathematical model. Modrzejewska et al. [35] proposed the sorption kinetic model, which combine chemical reaction and intraparticle diffusion. The chemical reaction was characterized by the rate constant (1.69 × 10^−5^ s^−1^), and by the diffusion coefficient (6.23 × 10^−10^ m^2^ s^−1^). This value is only slightly higher than the diffusion coefficient of chitosan (4.0 × 10^−10^ m^2^ s^−1^) determined on the basis of laser refraction experiment [36].

As described above, published studies are mainly focused on the adsorption processes [4,27,28,29,30,31]. Some of them deal with the diffusion through chitosan membranes and films [32,33,34,35]. Our approach is different. Since chitosan is considered as the material with high potential to immobilize toxic metal ions, it is desirable to investigate the interactions in detail. This study is focused on the reactivity mapping of chitosan in combination with the passing of diffusing metal ions through material containing chitosan as the active substance. In order to distinguish between the diffusivity through reactive and non-reactive medium, agarose hydrogel was chosen as basic non-reactive material. Agarose hydrogel proved to be the suitable medium for the investigation of diffusion of different substances. [37,38,39]. It can be enriched by active substance which can interact with diffusing particles and partially immobilize them. Therefore, the interactions of diffusing metal ions with active substance incorporated in the hydrogel can be studied in their motion. Thus, the main aim of this study is the detailed analysis of these two parallel processes.

## 2. Results and Discussion

### 2.1. Diffusion Experiments

The main goal of this study was to investigate the effect of chitosan on the mobility of copper(II) ions in agarose hydrogel. The diffusion experiments presented in this paper follow previous works which investigated the barrier effects of other biopolymer-humic acid on the transport of copper(II) ions by means of different methods [37,38,40,41,42]. We expected a similar effect of chitosan on the migration of metal ions as in the case of adding humic acids to hydrogel caused by interactions between copper(II) ions and biopolymer because of its chelating ability. Interactions of chitosan with metal ions can be realized due to its hydroxyl and amino groups, which participate in their adsorption processes [4,12,16,17,23,35]. The functional groups represent coordination and binding sites in chitosan structure. In general, other approaches to interactions between copper(II) ions and chitosan can be discussed. The first approach proposes the coordination site formed by four amino groups, another considers two amino and two hydroxy groups [16]. The chelation can be also realized by means of functional groups from the same chitosan chain, different chitosan chains, or by one amino group and one hydroxy group in coordination with two water molecules [12,43,44]. The ion exchange can participate in the interactions of chitosan with metal ions [26,45].

**Figure 1:**
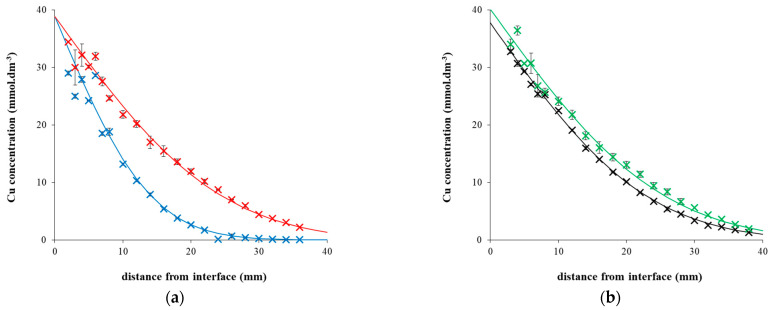
(**a**) Concentration profiles of copper(II) ions in agarose hydrogel enriched by chitosan (0.5 mg g^−1^) after 24 h (blue) and 72 h (red). (**b**) Concentration profiles of copper(II) ions in pure agarose hydrogel (black) and hydrogel enriched by chitosan (1 mg g^−1^; green) after 72 h. Experimental data are fitted by Equation (1).

In Figure 1a, the concentration profiles of copper(II) ions in hydrogel enriched by chitosan (0.5 mg g^−1^) is shown two different times. The increase in concentration (at a given distance from the interface) with prolongation of diffusion experiments was observed. A similar increase in the time development of concentration profiles was observed for pure agarose hydrogel. In Figure 1b, the comparison of concentration profiles for pure agarose hydrogel and hydrogel enriched by 1 mg g^−1^ of chitosan is shown. The concentration profiles were fitted by a mathematical model based on Fick’s laws [46,47,48] and the following assumptions. In beginning, the cuvettes with hydrogel (free of copper(II)ions) were covered by the donor solution with initial concentration *c*_0_. Hydrogels can be considered as semi-infinite mediums. It means that the hydrogel closed to bottom of cuvette remained free of copper(II) ions during whole diffusion experiment. The concentration changes of diffusing metal ions at the interface between hydrogel and donor solution are negligible (the maximal calculated decrease in the amount of copper(II) ions in donor solution is 0.5 %). It means that a concentration at the interface *c_s_* can be considered as constant. The assumptions can be expressed as initial and boundary conditions in Table 1. In the case of non-reactive hydrogel, the time development of the concentration profile in hydrogel can be thus be expressed as Equation (1):

(1) $$c=c_{s}erfc\frac{x}{2\sqrt{D_{h}t}}$$

where *t* is time, *x* is distance from interface, *c* is concentration of copper(II) ions, *c*_s_ is concentration at interface, and *D_h_* is the diffusion coefficient of copper(II) ions in hydrogel [47,48]. The total diffusion flux *m_t_* then depends on the square root of time according to Equation (2):

(2) $$m_{t}=2c_{s}\sqrt{\frac{D_{h}t}{\pi }}$$

**Figure 2:**
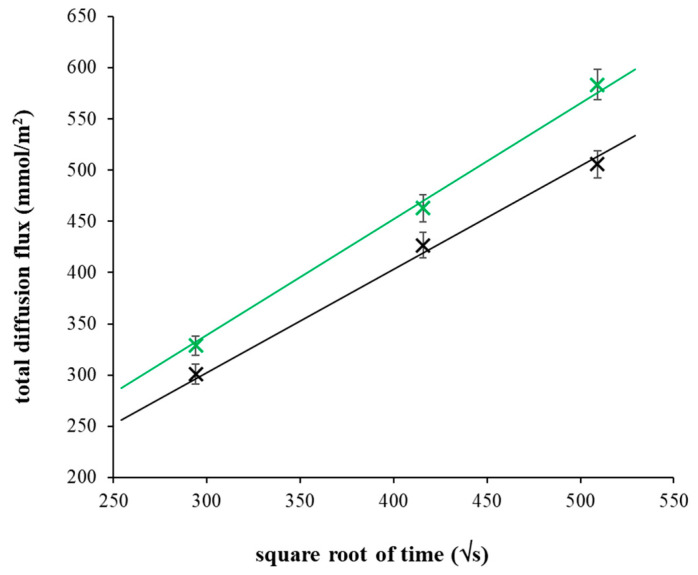
Experimental data fitting for the calculation of effective diffusion coefficients. The dependence of total diffusion flux of copper(II) ions in pure agarose hydrogel (black) and hydrogel enriched by chitosan (1 mg g^−1^; green) on square root of time. Experimental data are fitted by Equation (2).

Concentration profiles in Figure 1 are fitted by Equation (1). The diffusion coefficient used for the fitting was determined on the basis of Equation (2) from the slope of dependency shown in Figure 2.

The diffusion coefficient *D_h_* is valid for the diffusion in non-reactive hydrogel and its value is affected by the pore structure of hydrogel. If the particles diffuse in hydrogel, their motion is influenced by size of pore volume accessible for the diffusion. It can be expressed as the porosity *φ*; meaning that a portion of free volume joins the bulk volume of hydrogel. The diffusion in pores, which are not straight, also requires a longer diffusion path in comparison with the diffusion in homogeneous medium as, e.g., water [36,37,38,39]. The longer diffusion path can be characterized by tortuosity factor *τ* which is the ratio of squares of a real (effective) diffusion path in pore *L_ef_* and a macroscopic distance *L*:

(3) $$\tau ={\left(\frac{L_{ef}}{L}\right)}^{2}$$

Thus, the diffusion coefficient in non-reactive hydrogel can be calculated as the product of the structure factor of hydrogel (*μ*), and the diffusion coefficient of copper(II) ions in water (*D*):

(4) $$D_{h}=\mu D=\frac{\varphi }{\tau }D.$$

If the hydrogel is enriched by an active substance able chemically interact with diffusing particles, the determined value of diffusion coefficient also includes the influence of the interactions. If we assume a simple equilibrium between bound immobilized particles (concentration *c_im_*) and free movable particles (concentration *c_free_*), an apparent equilibrium constant *K* as the ratio between these two forms of particles can be included in the effective diffusion coefficient *D_ef_* [38,39,46]:

(5) $$D_{ef}=\frac{D_{h}}{\frac{c_{im}}{c_{free}}+1}=\frac{D_{h}}{K+1}=\frac{\mu D}{K+1}.$$

The symbol *D_h_* in Equations (1) and (2) can be replaced by the effective diffusion *D_ef_*, and the modified equations can be used in the case of the hydrogel containing active substance. The apparent equilibrium constant then can be calculated as:

(6) $$K=\frac{D_{h}}{D_{ef}}-1$$

The values of diffusion coefficients and concentration at interfaces for pure agarose hydrogels and hydrogels containing different amounts of chitosan are listed in Table 2. 

It can be seen that both quantities depend on the content of chitosan in hydrogel. The concentration of Cu(II) ions in the interface gradually increases from pure agarose hydrogel up to the hydrogel with the highest chitosan content. The dependence of diffusion coefficient on the chitosan content is more complicated. According to Equation (5), the effective diffusion coefficient determined for hydrogel enriched by an active substance should be lower than the diffusion coefficient of the same diffusing particles in non-reactive hydrogel and in water. All calculated diffusion coefficients in Table 1 are lower than diffusion coefficient of Cu(II) ions in water [49]. This means that the pore structure of hydrogel decelerated the diffusion rate as a result of smaller pore volume accessible for the diffusion and longer diffusion paths in comparison with the diffusion in homogeneous medium as, e.g., water [36,37,38,39]. On the other hand, interactions between Cu(II) ions and chitosan in more enriched hydrogels had opposite effect than it was expected.

**Figure 3:**
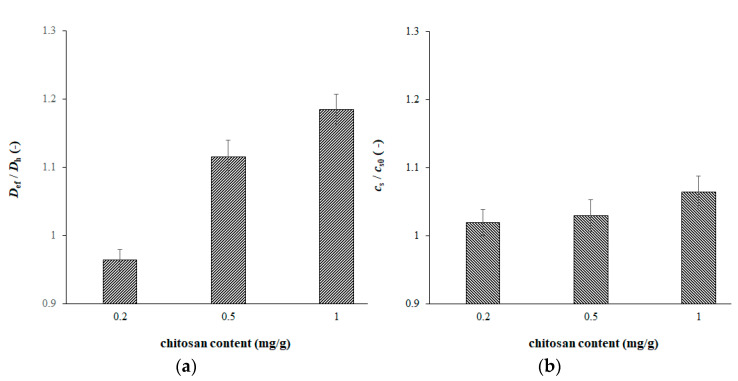
The ratios between effective diffusion coefficient *D_ef_* obtained for hydrogels containing chitosan and *D_h_* obtained for pure agarose hydrogel (**a**), and ratios between concentration of copper(II) ions at interface *c_s_* obtained for hydrogels containing chitosan, and *c_s_*_0_ obtained for pure agarose hydrogel (**b**).

The ratios between values obtained for hydrogels containing chitosan and pure agarose hydrogel are shown in Figure 3. We can see that the ratio of diffusion coefficients is less than 1 only for the lowest chitosan content; other ratios are higher than 1. Effective diffusion coefficient of copper(II) ions in hydrogels enriched by chitosan were determined in the range of (6–7.5) × 10^−10^ m^2^ s^−1^. The values obtained in this work are similar to the diffusion coefficient determined for copper(II) ions in water (7.1 × 10^−10^ m^2^ s^−1^) [48,49]. Kyzas et al. [48] determined diffusion coefficients of copper(II) ions in cross-linked chitosan and grafted chitosan derivatives with poly(acrylamide) and poly(acrylic acid). They used the model of swollen spherical particles combining adsorption and diffusion processes. Obtained values were 3.64 × 10^−10^ m^2^ s^−1^, 2.52 × 10^−10^ m^2^ s^−1^, and 9.88 × 10^−10^ m^2^ s^−1^. Their results were in the same magnitude as our values. The authors also observed an increase in the diffusivity in chitosan grafted with poly(acrylic acid) (comparing with the diffusion in water). Zhang et al. [50] published diffusion coefficients in hydrogels used in DGT and DET techniques. The values were 6.20 × 10^−10^ m^2^ s^−1^ for pure agarose hydrogel and 6.38 × 10^−10^ m^2^ s^−1^ for water. The diffusion coefficient of copper(II) ions in hydrogel beads based on chitosan was determined as 6.23 × 10^−10^ m^2^ s^−1^ [35]. Thus, our results agree with some previous works and determined diffusion coefficients have values characteristic for hydrogel samples. In contrast, our results do not agree with hypothesis expressed by Equations (5) and (6), which include a simple equilibrium between immobilized and free movable particle.

Thus, an explanation can be found in the more complex mechanism of interactions between copper(II) ions and chitosan. The affinity of chitosan to metal ions is well-known. Published studies are focused mainly on adsorption batch experiments, e.g., [17,19,25,27]. Some works combined adsorption models with intraparticle diffusion [4,35,51] or deals with the diffusion through chitosan membranes and films [8,34]. Our approach is different. We are focused on the interaction directly realized in the motion of copper(II) ions in hydrogel containing chitosan as an active substance. The mechanism of interactions is relatively complex, and influenced by many factors as heterogeneity of chitosan and ionic environment of aqueous solution. 

As mentioned above, the interactions of chitosan with metal ions include adsorption, ion exchange, and chelation. The most important reaction and coordination sites are amino and hydroxyl groups [4,31,35,51,52]. The interaction between metal ions and chitosan is comprised of more reaction steps for the protonation or dissociation of functional groups, formation of chelate rings, and others. Modrzejewska [52] described several models of copper-chitosan complexes, and discussed its probability, binding possibility, and structure. On the basis of her own data and published results, she stated that the complexation of copper(II) ions is more effective for oligomers, and a polymerization degree is required. She proposed mechanisms of complexation for different functional groups of chitosan (NH_2_ and OH). Guzman et al. [53] pointed to many differences in the abundant literature about metal binding on chitosan. They stated that an important underestimated parameter of the binding mechanism is the metal ion speciation (affected by pH). Similarly, this influence was discussed by Guibal et al. [27,28]. Published results showed that the interactions between chitosan and copper(II) ions cannot be described by a simple equilibrium between immobilized and free movable metal ions, and an apparent equilibrium constant *K* in Equations (5) and (6). The mechanism not only includes one reversible reaction, but it can be comprised of more consecutive reactions in the interactions can form more intermediates and final products (including, e.g., eliminating of ions). Nevertheless, the ratio between the diffusion coefficient in pure agarose hydrogel (*D_h_*), and the hydrogel enriched by chitosan (*D*_ef_) represents the important characteristics of interactions and their influence on the mobility of metal ions in hydrogel.

**Figure 4:**
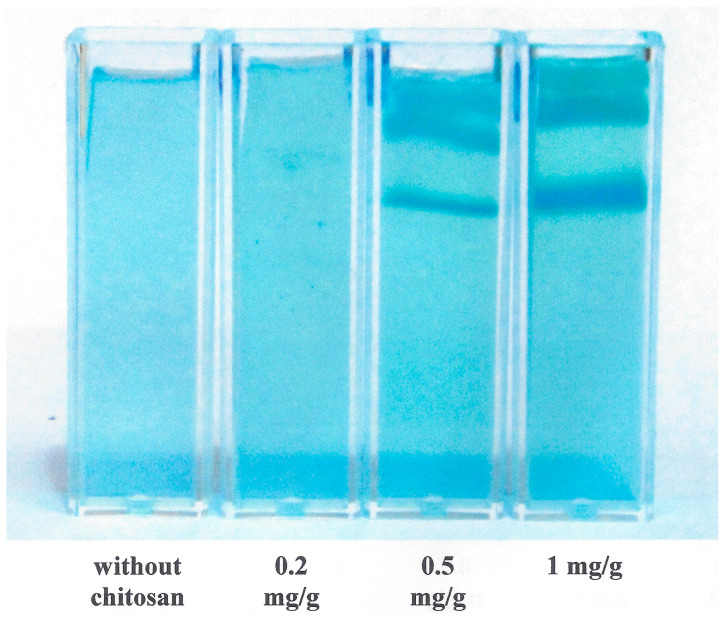
Formation of Liesegang patterns in agarose hydrogel enriched by chitosan.

In addition, the interaction of copper(II) ions with chitosan contained in the hydrogel can result in insoluble or slightly soluble products. The products can be observed as colored layers shown in Figure 4. We can see that the Liesegang patterns [54,55,56] are stronger with higher amount of chitosan in hydrogel. In contrast, no colored layers were observed in pure agarose hydrogel. The Liesegang phenomena are probably the reason why the measuring error of concentration profiles measured in hydrogels enriched in chitosan is higher in the region closed to the interface (Figure 1). The formation of Liesegang structures in systems containing chitosan was studied in several works [57,58,59,60]. The Liesegang pattern occurs when a precipitation reaction is coupled to the mass transport of reagents in hydrogel [61]. The phenomenon is usually described by means of the spacing law assuming that the precipitate zones are members of a geometrical series and the time law expressing that the zones are directly proportional to square root of time [56,57,60]. Simultaneously, the chemical mechanism is described by simple model comprised of two consecutive reaction steps: the formation of soluble intermediate (I), and its transformation to precipitate (P): A_(aq)_ + B_(aq)_ → I_(aq)_ → P_(s)_. Although this model is widely used, the mechanism is not able to express the mechanism of interaction between metal ions and chitosan. The first “imperfection” of the model is an absence of an equilibrium between precipitated and dissolved forms of metal-chitosan complexes. The periodic precipitation occurs when particles (ions) dissolved in water diffuse through hydrogel and can form a weakly soluble substance (complex, salt) with other substance containing in hydrogel. The hydrogel medium is important to prevent the sedimentation of formed precipitate [61]. In the case of ionic reaction, the soluble salt is formed in the initial step as the intermediate, and its transformation to precipitate proceeds if the product of concentrations of reacting ions (e.g., [A^+^] × [B^−^]) exceeds the so-called solubility product *K*_sp_ [61,62]. The solubility product represents the simplest theoretical approach to the weakly soluble substances. It considers precipitate as salt, which can partially dissociate: AB_(s)_ ↔ A^+^_(aq)_ + B^−^_(aq)_. The solubility product is then defined as the product of activities of both ions in its saturated solution. The activities can be (under certain circumstances) replaced by their concentrations [63]:

(7) $$K_{sp}=a_{A^{+}}.a_{B^{-}}≅\left[A^{+}\right]\left[B^{-}\right]$$

The value of *K_sp_* is constant under given temperature and pressure. The solubility product is in fact a special type of equilibrium (dissociation) constant. Its value includes only activities (concentrations) of dissociated ions because the chemical potential of solid phase is constant and can be included in the equilibrium constant *K_sp_*. This is formally expressed as its unit activity. In the case of dilute solution, the activities equal to concentrations can be supposed.

The described simple dissociation model thus represents the reversible reaction characterized by the equilibrium constant *K_sp_*. As mentioned above the model of chemical mechanism of Liesegang phenomena comprised of two consecutive reaction steps is lacking in an equilibrium between dissolved particles and precipitate. On the other hand, the equilibrium between solid salt and their dissolved ions is lacking in a soluble intermediate, and is too simple to characterize the Liesegang phenomenon, and the interactions between copper(II) ions and chitosan in studied hydrogels. Nevertheless, our findings showed that it is necessary to include reverse reactions and equilibrium between solid and dissolved particles into the reaction mechanism. In general, the above-described mechanism can be modified in the model of two consecutive reaction steps, which are both reversible reactions: A_(aq)_ + B_(aq_) → I_(aq)_ → P_(s)_. Both reaction steps can be characterized by its own equilibrium constant. This model can be re-written in the ionic form as: A^+^_(aq)_ + B^−^_(aq)_ ↔ AB_(aq)_ ↔ AB_(s)_. A direct equilibrium between precipitate and dissociated ions is not involved here. It is an open-ended question if the solid precipitate can dissociate and form dissolved ions without the formation of dissolved intermediate. It was reported that some precipitation reactions are not governed by solubility product *K_sp_*, but by the threshold of aggregation, which is related to the nucleation threshold concentration of AB_(aq)_ intermediate (c*) [61,62,64]. One example is the reaction system, where the precipitate can be dissolved in an excess of one reagent. In these systems, the mechanism also consists of two consecutive reaction steps: the precipitation A_(aq)_ + B_(aq)_ → P_(s)_, and the dissolution of precipitate accompanied by the formation of its complex with the dissolved reagent P_(s)_ + A_(aq)_ → P-A_(aq)_. This type of reaction proceeding diffusion through hydrogel can result in a seemingly moving precipitation zone [64,65].

The combination of precipitation reaction with mass transport of a reagent through hydrogel containing active substance can lead to more complex reaction mechanism involving reverse reactions and potential redissolution of precipitate. In the case of known reaction mechanism, we can modify Fick’s law and add the reaction rate *r* into the equation for non-stationary diffusion.

(8) $$\frac{\partial c}{\partial t}=D_{h}\frac{\partial^{2}c}{\partial x^{2}}+r$$

Then, we can express the rate of decrease in the concentration of diffusing particles according to the reaction mechanism. In the simplest case (reaction A + B without reverse reaction), the reaction rate *r* can be expressed as the product of rate constant *k*, concentration of diffusing particles and concentration of active substance in hydrogel. The problem is that Equation (8) is not directly (analytically) soluble. An analytical solution can only be obtained for the simple equilibrium between free movable and immobilized particles (see above). As it was found, our results are not in agreement with this simple model. Some authors derived more complex mathematical models for the description of Liesegang phenomenon, and used numerical methods for their solution [56,61,64]. Iszák and Lagzi [56] suggested a new universal model for the formation of Liesegang patterns based on the time dependent quantities of the precipitate. Saad et al. [64] stated that Liesegang systems with re-dissolving reaction exhibit deterministic chaos in the dynamic oscillations of the numbers of bands. Nabika et al. [61] reviewed existing models of the Liesegang phenomenon including different pre- and post-nucleation scenarios. They discussed also the model including three consecutive processes (reaction, nucleation, and aggregation): A_(aq)_ + B_(aq)_ → I_(aq)_ → I_(s)_ → P_(s)_. The model calculates with three different “threshold” constants, rate constants, and diffusion coefficients.

As can be seen, the situation in the hydrogel systems in which the diffusion is accompanied by the chemical reaction and precipitation is complicated. In most cases, suggested mechanisms and models involved more consecutive steps without reverse reactions. In contrast, they assumed a supersaturation or exceeding of the solubility product and different “threshold” constants which can be considered as equilibrium constants. The aim of this study is not to solve the mechanism of Liesegang phenomenon; it is discussed in connection with the effective diffusion coefficient. In beginning, we assumed the simple equilibrium between free diffusing copper(II) ions and their immobilized form, but this equilibrium should lead to the decrease in diffusion coefficient, which was not confirmed. It is a question if the decrease in diffusion coefficient obtained for hydrogel containing the lowest amount of chitosan means a different mechanism of Liesegang phenomena. According to Equation (6), the apparent equilibrium constant *K* should be equal to 3.72 × 10^−2^. It is relatively small value showing on very low degree of immobilization. Since the donor solutions with the same initial concentration were used in our experiments, the differences between diffusion coefficients determined for pure and enriched hydrogels should be caused by the different content of chitosan. 

Some published models of Liesegang mechanism assume the re-dissolving of precipitate caused by an excess of diffusing ions. In contrast, the content of active substance (chitosan) incorporated in hydrogels changed in our experiments obtained a “break” in the ratio between *D_ef_* and *D_h_* (see Table 2 and Figure 3); thus, it probably cannot be caused by changing the mechanism from a simple immobilization equilibrium into more complex mechanism involving the re-dissolution. As mentioned above, we can define two deficiencies: an absence of reverse reactions (and related equilibriums) in proposed mechanisms, and too many parameters in published models, which can only be solved numerically. The other problem is that our results cannot be explained by the re-dissolving because the diffusivity of metal ions is dependent on the content of chitosan “fixed” in hydrogel. We have also to take into consideration that the decrease in diffusion coefficient is not necessarily connected with the decrease in the diffusion rate. The rate of diffusion is strongly dependent on the concentration gradient, and it increases with the immobilization of metal ions. The reason is that if the copper(II) ion is immobilized by chitosan, it disappears from the solution and cannot participate on the diffusion. The resulting diffusion rate is a combination of both influences. Although the effective diffusion coefficient *D_ef_* cannot by simply expressed by means of the apparent equilibrium constant *K*, we believe that its value can be considered as a parameter characterizing degree of interactions between diffusing metal ions and chitosan as active substance incorporated in hydrogel. In the given conditions, a more precise definition of *D_ef_* is not possible. Different published models and mechanisms require numerical methods for solution and modified Fick´s law, Equation (8), together with initial and boundary conditions, defined on the basis of the experimental arrangement and selected mechanism, cannot provide an analytical solution. Therefore, we are not able to directly express *D_ef_* as a function of parameters characterizing proceedings chemical reactions (equilibrium and rate constants).

### 2.2. Rheological Experiments

The diffusion of metal ions in agarose hydrogel is influenced by two basic factors: their interactions with chitosan as active substance (if incorporated), and the structure of hydrogel. It is not easy to distinguish between the effect of hydrogel structure and the effect of their reactivity. It is well-known that diffusivity of spherical particles is dependent on their size (radius *r*) and viscosity of the diffusion medium *η* [46,47,63,66]:

(9) $$D=\frac{k_{b}T}{6\pi \eta r}$$

where *k_b_* is Boltzmann constant and *T* is temperature. Equation (9) can be used in the case of Newtonian liquid medium characterized by one value of dynamic viscosity *η* at given temperature *T*. Rheological behavior of hydrogels is usually more complex [3,37,66]. They can be considered as viscoelastic substances combining behavior of viscous liquids and elastic materials. Therefore, their rheological behavior cannot be characterized by one value of viscosity. Our rheological measurements provided information about the viscoelasticity of the studied hydrogels and changes caused by the addition of chitosan.

**Figure 5:**
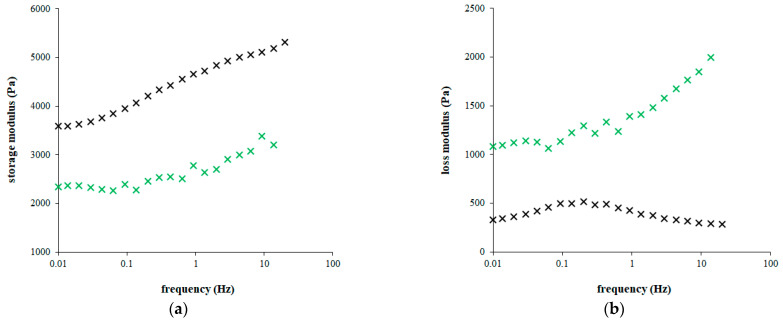
Storage modulus (**a**) and loss modulus (**b**) measured for pure agarose hydrogel (black) and hydrogel enriched by chitosan (1 mg g^−1^; green). Measurement was realized in linear viscoelastic region (strain 0.1 %).

Figure 5 shows the dependencies of the storage and loss moduli on the frequency. The strain (0.1 %) was chosen from linear viscoelastic region, where the moduli are strain independent. The storage modulus *G*’ proportional to the elastic properties increased with increasing oscillation frequency for both types of hydrogels. As can be seen, its values are much higher for pure agarose hydrogel. They decreased gradually with increasing content of chitosan (not shown) up to approximately 60% of their initial values. It means that the addition of chitosan resulted in more liquid character of hydrogel and the pure agarose hydrogel is the most resistant to mechanical stresses. The frequency dependencies of loss modulus *G*´´ had different shapes. While its values measured for hydrogel containing chitosan are approximately constant up to 0.1 Hz, then strongly increases, the dependence obtained for pure agarose hydrogel had a flat maximum around 0.2 Hz. 

**Figure 6:**
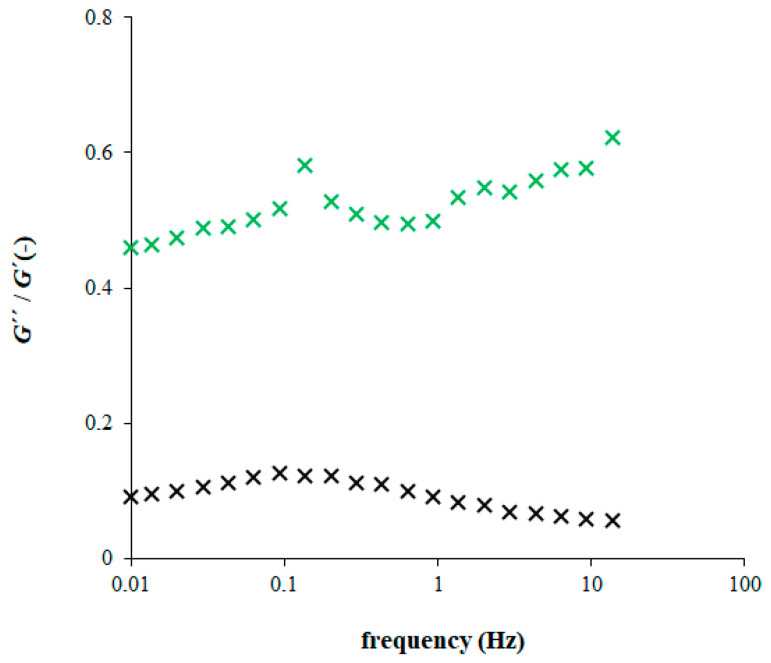
The ratio between loss (*G*″) and storage (*G*′) moduli (pure agarose hydrogel–black; hydrogel enriched by chitosan (1 mg g^−1^)-green).

Values obtained for pure agarose hydrogel are much lower in comparison with hydrogels containing chitosan which confirmed its high resistance to stresses. The frequency dependence of ratio between both moduli had, for pure agarose hydrogel, am identical shape as the loss modulus (Figure 6). A maximum was also observed for hydrogel enriched with chitosan. The “most liquid character” of chitosan was achieved at lower oscillation frequency 0.14 Hz. In contrast to pure agarose hydrogel, the ratio then decreased up to 0.63 Hz, and increases at higher frequencies. The highest value of *G*″/*G*′ ratio was comparable with the maximum. The complex viscosity was lower for hydrogels containing chitosan and decreased gradually with increasing oscillation frequency for all studied hydrogels.

**Figure 7:**
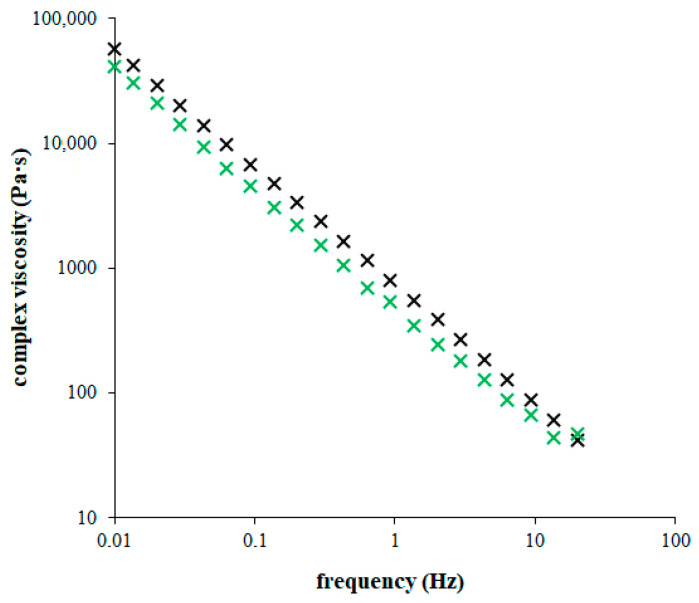
Complex viscosity measured for pure agarose hydrogel (black) and hydrogel enriched by chitosan (1 mg g^−1^; green). Measurement was realized in linear viscoelastic region (strain 0.1 %).

There was no cross-point of storage and loss moduli was observed, and the elastic character predominated at all frequencies for all studied hydrogels. Our results showed that the rheological behavior of hydrogels was changed by the addition of chitosan. The behavior of enriched hydrogels shifted gradually to more liquid character as the chitosan content increased. There was no break in rheological properties when its addition was observed. All changes were gradual. As can be seen, the complex viscosity decreases with frequency gradually, the dependence is linear in logarithm axis (Figure 7). This means that the changes in rheological behavior of hydrogels cannot directly result in the break in the diffusivity of copper(II) ions described above. On the other hand, the transport properties of hydrogels were affected by a combination of two influences–rheological character of hydrogels and their reactivity (if contained chitosan as active substance). The hydrogels containing chitosan had lower abilities to resist mechanical stresses, which can be connected with their higher permeabilities. This effect can prevail over the effect of interactions between chitosan and copper(II) ions. The shifting to more liquid character for hydrogels containing chitosan can resulted in their higher permeability connected with higher effective diffusion coefficients obtained for more enriched hydrogels. The effect of chemical interactions in enriched hydrogels was strong and visually observable. On the other hand, the contents of chitosan in hydrogels were low. It means that the amounts of active sites in hydrogels were limited and the portion of copper(II) ions which can by immobilized. According to the value of apparent equilibrium constant *K* determined in previous chapter, only less than 4% of copper(II) ions can be immobilized.

## 3. Conclusions

Despite the contents of chitosan in the hydrogels were very low, its addition resulted in changes in their viscoelasticity and permeability. The changes were influenced by two contrary effects. Although the storage modulus was higher than the low one and the elastic character predominated for all studied hydrogels, the addition of chitosan caused the hydrogels to become more liquid, and, therefore, more permeable for diffusing particles. In contrast, the interactions of copper(II) ions in chitosan and formation of precipitate caused the decrease in free movable ions in hydrogel and changes in the effective diffusion coefficient. The value of *D_ef_* included both the influence of hydrogel structure and the effect of immobilization of copper(II) ions. These contrary effects influenced final value of diffusivity in varying degrees according to the given content of chitosan. Its low content resulted in the decrease in the value of *D_ef_*. We can believe that the effect of immobilization of copper(II) ions prevailed above the changes in theological character of hydrogel and its “more liquid character” can be considered less strong. Higher amounts of chitosan in hydrogels shifted their rheological behavior more to liquid, and the permeability of hydrogels were much higher than the effect of chemical interactions between copper(II) ions and chitosan. Although the simple model of the equilibrium between immobilized and free copper(II) ions cannot express the mechanism of the interactions, we can state that the portion of immobilized metal ions is relatively low. It means that the possibility to immobilize metal ions is limited by the content of chitosan. The diffusion of copper(II) ions in hydrogels enriched by chitosan can thus be accelerated by the more liquid character of hydrogel, and the increase in concentration gradient or the diffusion, which can be suppressed by the decrease in the diffusion coefficient as the effect of chemical reaction.

## 4. Materials and Methods

### 4.1. Chemicals

Chitosan (medium molecular weight), agarose (routine use class) and copper(II) sulfate (p.a.) were purchased from Sigma Aldrich (St. Luis, MO, USA). Acetic acid for the preparation of chitosan solution was purchased from Lachner (Neratovice, Czech Republic). 

The exact molecular weights of chitosan and agarose were determined by means of size exclusion chromatography, coupled with multiangle static light scattering, differential refractive index, and UV/VIS detection (SEC chromatographic system from Agilent Technologies, detectors from Wyatt Technology). The exact molecular weights were 251 ± 4 kDa for chitosan and 146 ± 3 kDa for agarose.

Deacetylation degree of chitosan was determined by potentiometric titration described by Garcia et al. [67]. The degree was determined as 83.8 ± 0.2% mol.

### 4.2. Preparation of Hydrogels

The preparation of hydrogels was based on the thermo-reversible gelation of agarose solution described in previous works [36,37,38,39,66]. Agarose hydrogel gelatinized from the solution of agarose in water. Agarose content in hydrogel was 10 mg g^−1^. The mixture was slowly heated with continuous stirring up to 80 °C, stirred at this temperature order to obtain a transparent solution, and finally sonicated (1 min) to remove gasses. Afterwards, it was slowly poured into the PMMA spectrophotometric cuvette (inner dimensions: 10 × 10 × 42 mm). The cuvette orifice was immediately covered with a pre-heated plate of glass to prevent drying and shrinking of gel. The flat surface of the boundary of resulting hydrogels was provided by wiping an excess solution away. Gentle cooling of cuvettes at the laboratory temperature led to the gradual gelation of the mixture.

Agarose-chitosan hydrogels were prepared from agarose solution mixed with the solution of chitosan. An accurately weighed amount of chitosan was dissolved in 50 cm^3^ of acetic acid (5% wt.). The solution was titrated by 1M NaOH up to pH equal to 7 and diluted by distilled water (the final volume was 100 cm^3^). Agarose content in hydrogel was 10 mg g^−1^, the contents of chitosan were 0.2, 0.5, and 1 mg g^−1^, respectively.

### 4.3. Diffusion Experiments

Three cuvettes containing the same type of hydrogel were placed into 300 cm^3^ of donor solution (0.1 M CuSO_4_). The solution was stirred continuously by the magnetic stirrer and the copper(II) ions were left to diffuse from the solution into the hydrogels through the square orifices of the cuvettes. Diffusion experiments were triplicated, meaning that three different vessels for the same type of hydrogel (nine cuvettes in total) were used. The durations of the diffusion experiments were 24, 48, and 72 h, respectively. In these time intervals, the cuvettes were taken out of the solution, and the UV-VIS spectra were measured in dependence on distances from the interface between hydrogel and donor solution. The cuvettes were taken out of the solution and the spectra were measured on Varian Cary 50 UV–VIS spectrophotometer (Agilent Technologies, Palo Alto, CA, USA) equipped with the special accessory providing controlled fine vertical movement of the cuvette in the spectrophotometer [38,39]. Concentration of the copper(II) ions was determined at different positions in the hydrogels by means of calibration line. The spectra were calibrated for the hydrogels with the known concentration, homogeneously distributed in the whole volume of the hydrogel.

All experiments were performed at laboratory temperature (25 ± 1 °C). Data are presented as average values with standard deviation bars. 

### 4.4. Rheological Experiments

The hydrogels were sliced to obtain cylindrical samples suitable for rheological measurements (1 mm in thickness). Each hydrogel sample was placed between two titanium plates (40 mm in diameter) of an AR-G2 rheometer (TA Instruments, Ltd., New Castle, DE, USA) equipped with Rheology Advantage Instrument Control AR software. (v5.5.24, TA Instruments, Ltd., New Castle, DE, USA). Silicon oil was used to prevent drying of the hydrogels. The hydrogels were left to relax for 15 min before measurements were made. Measurements were performed at 25 ± 1 °C. Rheological behavior of hydrogels was characterized with respect to their viscoelastic character. The storage modulus *G*′ (proportional to the extent of the elastic component), loss modulus *G*″ (proportional to the extent of the viscous component), and complex viscosity η* were determined. All experiments were triplicated, and average values are presented.

## Figures and Tables

**Table 1:** Initial and boundary conditions of diffusion experiments.

Time *t*	Distance *x*	Concentration *c*
*t* = 0	*x* > 0	*c* = 0
*t* > 0	*x* = 0	*c* = *c_s_*
*t* > 0	*x* → ∞	*c* = 0

**Table 2:** Values of concentration of copper(II) ions at interfaces and diffusion coefficients determined for studied hydrogels.

Chitosan Content (mg g^−1^)	Concentration at Interface (mmol dm^−3^)	Diffusion Coefficient (m^2^ s^−1^)
0	37.73 ± 0.62	(6.25 ± 0.06) × 10^−10^
0.2	38.48 ± 0.86	(6.03 ± 0.13) × 10^−10^
0.5	38.86 ± 1.15	(6.98 ± 0.24) × 10^−10^
1	40.15 ± 1.12	(7.41 ± 0.22) × 10^−10^
